# Focal calcium monitoring with targeted nanosensors at the cytosolic side of endoplasmic reticulum

**DOI:** 10.1080/14686996.2016.1190258

**Published:** 2016-07-18

**Authors:** Yanyan Hou, Satoshi Arai, Yoshiaki Takei, Atsushi Murata, Shinji Takeoka, Madoka Suzuki

**Affiliations:** ^a^ Waseda Bioscience Research Institute in Singapore (WABIOS), Singapore, Republic of Singapore; ^b^ Department of Life Science & Medical Bioscience, Faculty of Science & Engineering, Waseda University, Tokyo, Japan; ^c^ Organization for University Research Initiatives, Waseda University, Tokyo, Japan; ^d^ PRESTO, Japan Science and Technology Agency, Saitama, Japan

**Keywords:** Copper-free click reaction, calcium imaging, nanosensor, N3-fura-2, endoplasmic reticulum, 30 Bio-inspired and biomedical materials, 208 Sensors and actuators, 101 Self-assembly/Self-organized materials

## Abstract

Ca^2+^ distribution is spatially and temporally non-uniform inside cells due to cellular compartmentalization. However, Ca^2+^ sensing with small organic dyes, such as fura-2 and fluo-4, has been practically applied at a single cell level where the averaged signal from freely diffusing dye molecules is acquired. In this study, we aimed to target azide-functionalized fura-2 (N_3_-fura-2) to a specific site of subcellular compartments to realize focal Ca^2+^ sensing. Using scAVD (single-chain avidin)–biotin interaction and a copper-free click reaction system, we linked N_3_-fura-2 to specifically-targeted scAVD protein fused with a red fluorescent protein mCherry, so that Ca^2+^ sensors conjugated with four N_3_-fura-2 dyes with dibenzocyclooctyne (DBCO)-PEG4-biotin as a linker were generated at subcellular compartments in living cells. In cytoplasm, N_3_-fura-2 showed a prolonged retention period after binding to scAVD. Furthermore, the reacted N_3_-fura-2 was retained inside cells even after free dyes were washed out by methanol fixation. When scAVD was overexpressed on endoplasmic reticulum (ER) membranes, N_3_-fura-2 was accumulated on ER membranes. Upon histamine stimulation, which increases cytosolic Ca^2+^ concentration, ER-localized N_3_-fura-2 successfully sensed the Ca^2+^ level changes at the cytosolic side of ER membrane. Our study demonstrated specific targeting of N_3_-fura-2 to subcellular compartments and the ability of sensing focal Ca^2+^ level changes with the specifically targeted Ca^2+^ sensors.

## Graphical abstract

**Figure:**
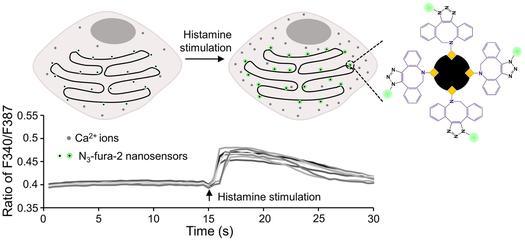

## 1. Introduction

Ca^2+^ signaling is crucial in maintaining cellular activities.[1] Due to cellular compartmentalization, Ca^2+^ is non-uniformly distributed between different organelles. Cellular organelles, such as endoplasmic reticulum (ER), mitochondria and Golgi complex, store Ca^2+^ and play fundamental roles in regulating Ca^2+^ levels through Ca^2+^ pumps and release channels on organelle membranes.[2] Monitoring the Ca^2+^ levels in those subcellular organelles as well as in the cytosolic regions that are in close vicinity to those organelles would provide direct evidence in investigating Ca^2+^ signaling pathways and all Ca^2+^-regulated cellular activities.

Calcium imaging at specific subcellular organelles has been generally performed by genetically encoded Ca^2+^ indicators.[3] Compared with those Ca^2+^ indicators, chemical Ca^2+^ indicators have a broader range of Ca^2+^ affinity with current commercially available repertoires.[4] Considering the non-uniformity of Ca^2+^ distribution inside cells, chemical Ca^2+^ indicators may have a wider application (e.g. to detect high Ca^2+^ concentration with a low affinity indicator and to detect low Ca^2+^ concentration with a high affinity indicator [4]). However, like most traditional organic dyes,[5] chemical Ca^2+^ indicators suffer from poor site-specificity and cannot be easily controlled to a particular organelle.[4]

**Figure 1.**
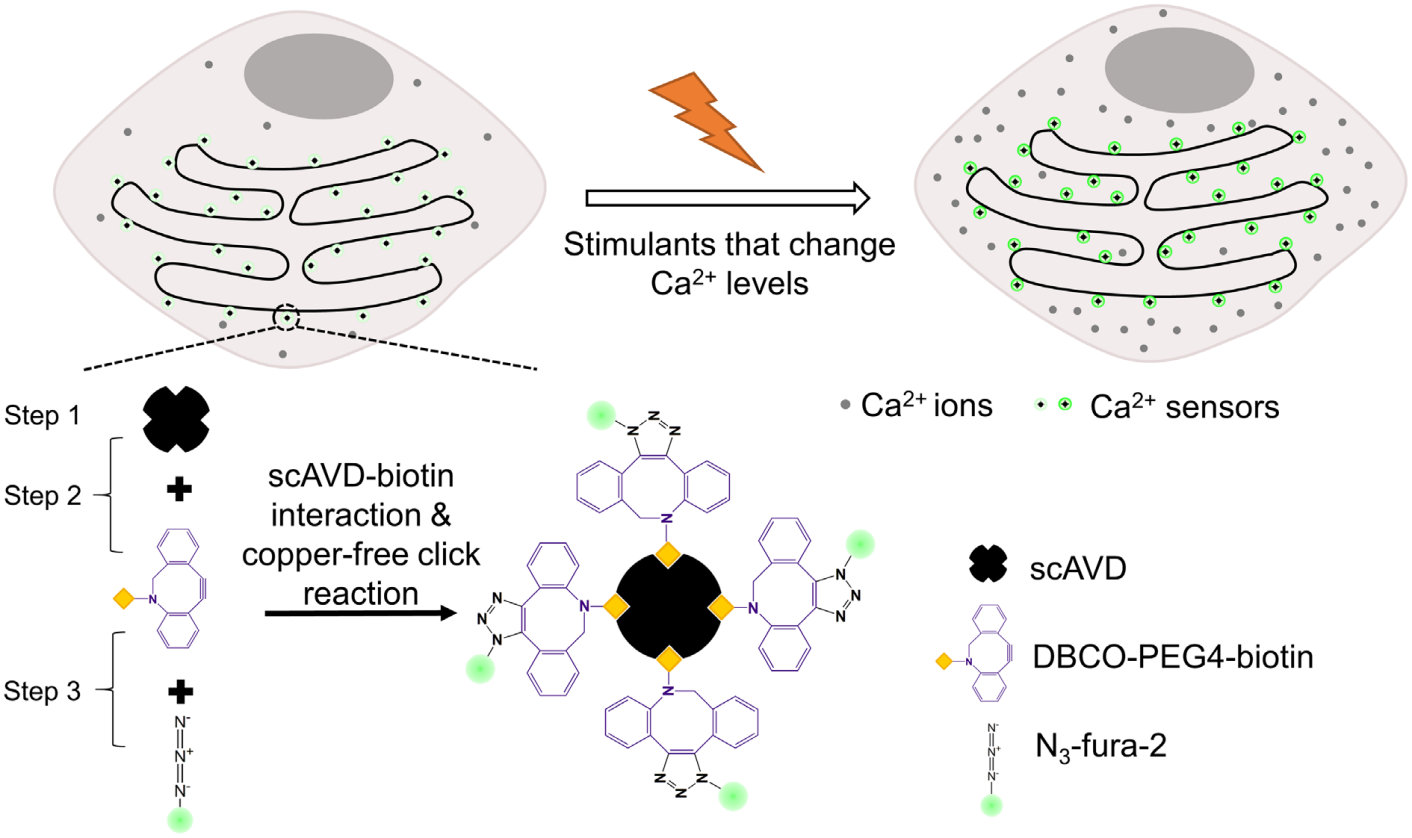
Schematic of monitoring focal Ca^2+^ concentrations with specifically targeted Ca^2+^ sensors at the cytosolic side of ER membrane. The four-fluorophore Ca^2+^ sensor was generated by three steps. Step 1: scAVD overexpression. Step 2: interaction between scAVD and DBCO-PEG4-biotin. Step 3: copper-free click reaction between DBCO and azide.

Specific and stable targeting of organic dyes can be realized by tagging organic dyes to proteins. The approaches include various tag-mediated protein labeling,[6,7] site-specific incorporation of unnatural amino acids,[8] and ligand-directed chemistry.[9] Using the tag-mediated labeling technology, several chemical Ca^2+^ indicators have been targeted to nucleus and cytosol in living cells.[10,11] Recently, we have reported a novel three-step method to specifically target organic dyes to intracellular organelles using scAVD–biotin interaction and bioorthogonal reaction between dibenzocyclooctyne (DBCO) and azide, so-called copper-free click reaction (Figure 1).[12] The first step of this method is to overexpress scAVD in living cells. As an advantage of the overexpression system, scAVD can target intracellular organelles by linking to an organelle-targeting sequence. As a tetravalent protein, scAVD is able to bind to four biotin molecules with high affinity.[13] This high affinity enabled efficient attachment of four DBCO-functionalized biotins in the second step. In the third step, azide-functionalized dyes reacted with DBCO by copper-free click reaction, creating a fluorescent probe conjugated with four fluorescent dyes (Figure 1). The probe exhibited increased brightness and enhanced photostability compared with single fluorescent dye.[12] Therefore, compared with the tag-mediated labeling technology which usually tags one fluorescent dye to a protein molecule, our method could generate probes with a higher signal to noise ratio, which is indispensable for single-molecule imaging. Using this method, we have targeted N_3_-Cy5 and N_3_-TAMRA dyes to the cytosolic side of ER where they tracked ER remodeling with single probe sensitivity and sensed ER temperature changes, respectively.[12]

In this study, we utilized this system to realize stable and specific targeting of N_3_-fura-2,[14] a ratiometric Ca^2+^ indicator, and to generate fluorescent Ca^2+^ sensors conjugated with four N_3_-fura-2 dyes. N_3_-fura-2 has a Ca^2+^ affinity of 304 nM (pH 7.2, 28°C) and a wide sensitivity range (~17 nM to ~39.8 μM).[14] Conjugation to macromolecules by click reaction does not significantly change its Ca^2+^ affinity.[14] The Ca^2+^ sensors conjugated with N_3_-fura-2 were targeted to both cytoplasm and ER membranes at the cytosolic side, for sensing focal Ca^2+^ concentration changes in response to stimulation (Figure 1).

## 2. Experimental details

### 2.1. Cell culture

Cells were cultured in Dulbecco’s modified eagle medium (DMEM) with 10% fetal bovine serum (FBS), 100 units ml^–1^ penicillin and 100 μg ml^–1^ streptomycin at 37°C in the presence of 5% CO_2_. All the components were purchased from Invitrogen (Carlsbad, CA, USA). Cells were cultured in 3.5 cm glass-based dishes (IWAKI, Tokyo, Japan) at 37°C with 5% CO_2_ for microscopy experiments.

### 2.2. Plasmids

scAVD DNA was synthesized by Integrated DNA Technologies (Coralville, IO, USA). mCherry-scAVD was generated by insertion of scAVD into a pmCherry-C3 vector. mSTIM1∆2-mCherry and mSTIM1∆2-mCherry-scAVD were generated by insertion of DNA fragment of mSTIM1∆2 (1–343 amino acids) amplified by polymerase chain reaction (PCR) from mouse brain cDNA into the pmCherry-C3 vector and the pmCherry-scAVD-C3 vector, respectively.

### 2.3. Materials

DBCO-PEG4-Biotin was purchased from Click Chemistry Tools (Scottsdale, AZ, USA). Histamine was purchased form Sigma-Aldrich (St Louis, MO, USA). N_3_-fura-2 AM containing an azide moiety and an acetoxymethyl (AM) ester were synthesized according to the procedures previously reported.[14] AM ester will be cleaved by cellular esterase after N_3_-fura-2 AM dyes enter cells. Therefore, N_3_-fura-2, instead of N_3_-fura-2 AM, was used when describing intracellular phenomena.

### 2.4. Epifluorescence microscopy

**Figure 2.**
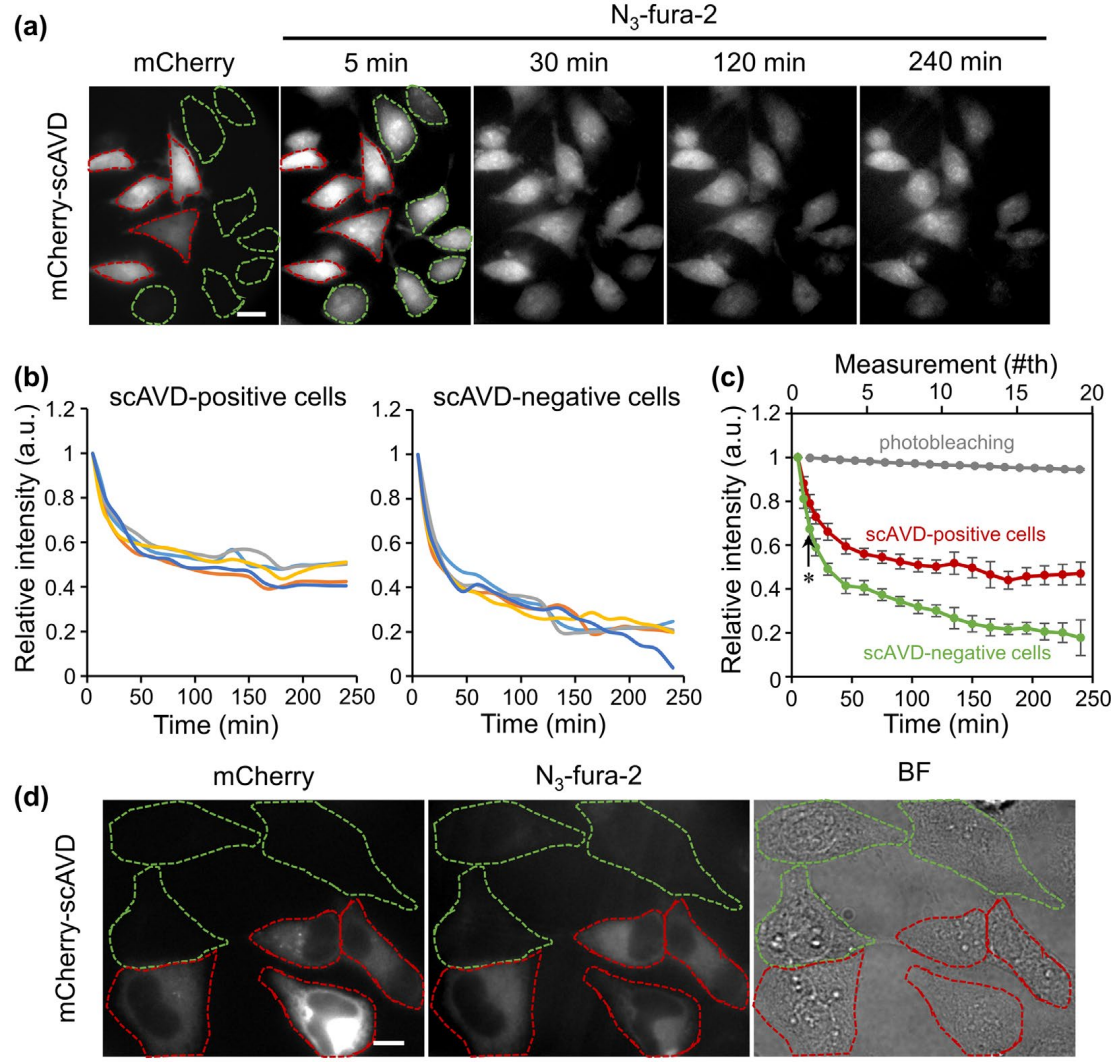
(a) Fluorescence images of HeLa cells transfected with mCherry-scAVD. Cells encircled with red and green dashed lines are cells overexpressing scAVD (scAVD-positive cells) and not overexpressing scAVD (scAVD-negative cells), respectively. Scale bar, 20 μm. (b) Fluorescence intensity changes of N_3_-fura-2 in scAVD-positive and scAVD-negative cells over 4 h. The fluorescence intensities of F387 were normalized to the first time point at t = 5 min. (c) Comparison of the average intensity of N_3_-fura-2 in scAVD-positive (red) and scAVD-negative (green) cells over 4 h (bottom axis). The photobleaching with the same exposure time was negligible (gray, top axis). Data are mean ± standard deviation (SD) (*n* = 5 in scAVD-positive and scAVD-negative cells; *n* = 8 in photobleaching test). Arrow points to t = 15 min. *, *p* = 0.004. (d) Fluorescence (left and middle) and bright-field (BF, right) images of HeLa cells fixed with methanol. Cells encircled with red and green dashed lines are cells overexpressing scAVD (scAVD-positive cells) and not overexpressing scAVD (scAVD-negative cells), respectively. Scale bar, 10 μm.

Cells were incubated with 10 μM DBCO-PEG4-biotin at 37°C for 1 h followed by incubation with 4 μM N_3_-fura-2 AM at 37°C for another 1 h in serum-free DMEM with 4-(2-hydroxyethyl)-1-piperazineethanesulfonic acid (HEPES) (Invitrogen). Cells were observed after three washes with phosphate buffered saline (PBS). When fixed cells were observed, cells were treated with 100% ice-cold methanol for 10 min at –20°C. Images were acquired with an Olympus IX83 inverted microscope equipped with an electron multiplying charge-coupled device (EMCCD) camera (iXon3, 1024 × 1024 pixels, Andor Technology, Belfast, UK). An UPlanSAPO 100× NA1.40 objective lens and an UPlanFL N 40× NA1.30 objective lens were used. A xenon burner (U-LH75XEAPO, Olympus) was used as a light source. mCherry images were acquired with an FF01-549/15 excitation filter, an FF585-Di01 dichroic mirror and an FF01-607/36 emission filter (all from Semrock, Rochester, NY, USA). N_3_-fura-2 images were acquired with FF01-340/26 (F340) and FF01-387/11 (F387) excitation filters, an FF409-Di03 dichroic mirror and an FF01-510/84 emission filter (all from Semrock). N_3_-fura-2 images excited at 387 nm wavelength (F387) were shown in figures. A MetaMorph NX software (Molecular Devices, Sunnyvale, CA, USA) was used to acquire images. In Figure 2(a), N_3_-fura-2 F387 images were captured with exposure time of 250 ms. In Figure 4, images were captured at 500 ms per frame with exposure time of 250 ms and 50 ms for N_3_-fura-2 F340 and F387, respectively.

### 2.5. Data analysis

Fluorescence images were analyzed with ImageJ software (National Institute of Health, Bethesda, MD, USA). In Figure 2(b), to calculate the relative intensity, the average fluorescence intensity of F387 of a whole cell at each time point was acquired and normalized to that of the first time point. In Figure 4(a), the average fluorescence intensity of F340 and F387 of a whole cell that overexpressed ER-targeting scAVD was acquired following histamine treatment. The ratio of F340 to F387 was then calculated to show the response profile to histamine. In Figure 4(b), images were shown after photobleaching correction with a CorrectBleach plugin [15]. The *p*-value shown in Figure 2(c) was calculated by Student’s *t*-test.

## 3. Results and discussion

To evaluate the intracellular binding ability of N_3_-fura-2, HeLa cells were transfected with cytoplasm-localized scAVD and subjected to incubation with DBCO-PEG4-biotin and N_3_-fura-2 AM in a sequential manner. Some cells were successfully transfected with mCherry (a red fluorescent protein)-tagged scAVD (Figure 2(a), cells encircled with red dashed lines), while others were not (Figure 2(a), cells encircled with green dashed lines). Cells were observed over 4 h to examine the amount of N_3_-fura-2 retained in scAVD-positive and scAVD-negative cells. As shown in Figure 2(a) and (b), N_3_-fura-2 quickly released from all cells in the first 1 h although the release rate in scAVD-positive cells was slower than that in scAVD-negative cells, indicating a portion of free N_3_-fura-2 dyes exist in scAVD-positive cells. After 1 h, N_3_-fura-2 continued to release out of scAVD-negative cells, while the level of N_3_-fura-2 in scAVD-positive cells reached a steady state (Figure 2(b)). Comparatively, scAVD-positive cells retained a higher level of N_3_-fura-2 than scAVD-negative cells over 4 h (Figure 2(c)), indicating a successful binding of N_3_-fura-2 to scAVD in living cells. At a low level, N_3_-fura-2 dyes were still present in scAVD-negative cells after 4 h. To remove the free N_3_-fura-2 dyes in cytoplasm, cells were fixed and permeabilized with ice-cold methanol. Methanol fixation dissolves lipids on cell membranes.[16] Therefore, after methanol fixation, the free dyes existing in the cytoplasm should be washed out. Indeed, scAVD-positive cells (Figure 2(d), cells encircled with red dashed lines) showed a much higher N_3_-fura-2 signal than scAVD-negative cells (Figure 2(d), cells encircled with green dashed lines) in which N_3_-fura-2 signal is hardly observable. This result further corroborates the notion that N_3_-fura-2 successfully bound to scAVD inside living cells by scAVD–biotin interaction and click reaction.

**Figure 3.**
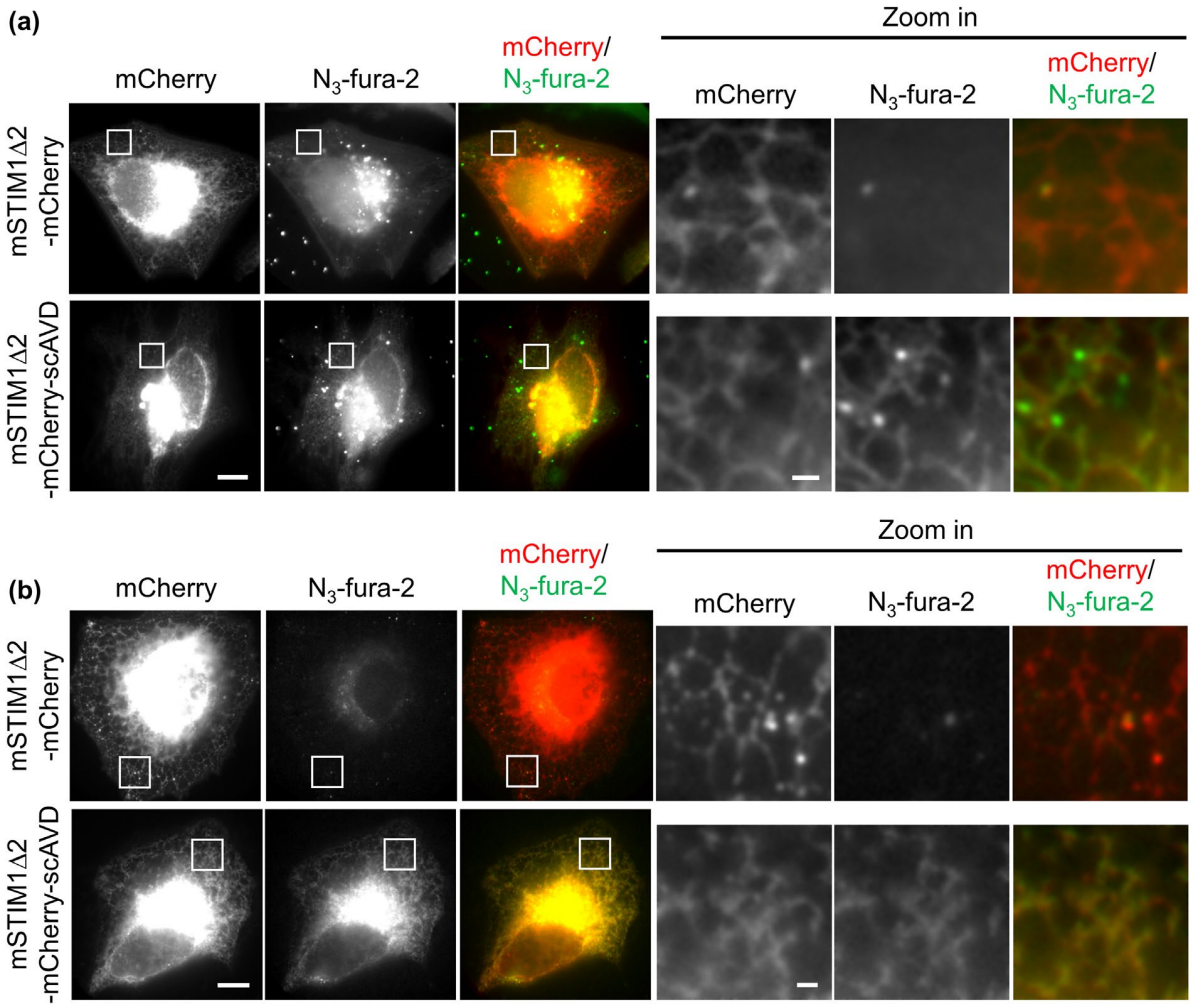
Fluorescence images showing colocalization of N_3_-fura-2 with ER in live (a) and fixed (b) scAVD-overexpressing HeLa cells. Regions in squares are zoomed in and shown in right panels. Scale bars, 10 μm for left panels, and 1 μm for right panels.

In order to target N_3_-fura-2 onto ER membranes at the cytosolic side, HeLa cells were transfected with an ER-targeting scAVD plasmid which contains a truncated mouse STIM1 fragment (mSTIM1∆2). The mSTIM1∆2 is an ER transmembrane protein with an N-terminus containing ER-targeting signal at ER lumen and a C-terminus at the cytosolic side.[12,17] The scAVD was fused to the C-terminus of mSTIM1∆2, creating a plasmid that targets scAVD to ER membranes at the cytosolic side. After incubation with N_3_-fura-2 AM, cells were washed with PBS and observed with a microscope. In vector cells, some bright N_3_-fura-2 aggregates were observed in the region close to nucleus, probably endocytosed N_3_-fura-2. In the remaining regions of the cytoplasm, a very low but evenly distributed N_3_-fura-2 signal was present, without accumulation on ER (Figure 3(a)). In contrast to vector cells, although bright N_3_-fura-2 aggregates were also present near the nucleus in scAVD-overexpressing cells, N_3_-fura-2 showed evident accumulation on ER in the remaining regions of the cytoplasm (Figure 3(a)), presented by a colocalization with ER-targeting scAVD. These results indicate the ER-localization of N_3_-fura-2 is due to the binding to scAVD. Fixation with methanol, which dissolves lipids not only on the plasma membrane but also on the endosomal membrane, led to a virtually complete removal of N_3_-fura-2 in vector cells, including both the free dyes in cytoplasm and the bright aggregates in endosomes (Figure 3(b)). By contrast, in scAVD-overexpressing cells, N_3_-fura-2 still remained on ER while bright aggregates disappeared (Figure 3(b)), suggesting the binding of N_3_-fura-2 to scAVD is stable.

**Figure 4.**
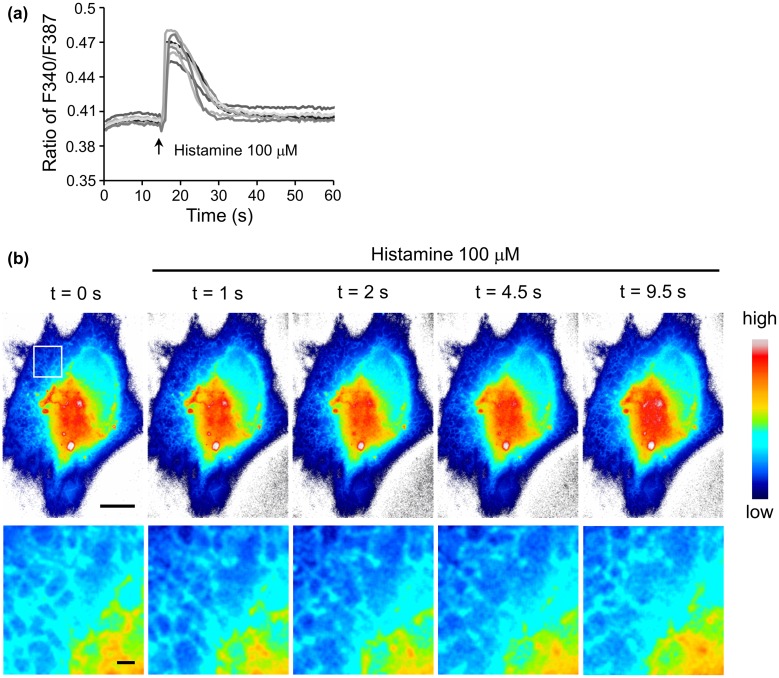
(a) Response of ER-targeting N_3_-fura-2 to histamine stimulation. The lines indicate the responses from seven individual cells. Fluorescence intensities of F387 and F340 were obtained from images acquired with a 40× objective lens. (b) Changes in fluorescence intensities F387 of ER-targeting N_3_-fura-2 in response to histamine stimulation. Intensities (after photobleaching correction) were shown as pseudo-color images. Images were acquired with a 100× objective lens. Region in square was zoomed in and shown in lower panel. Scale bars, 10 μm for the upper panel, and 1 μm for the lower panel.

Upon binding to ER, N_3_-fura-2 is likely to sense Ca^2+^ level changes in cytosolic regions that are in the close vicinity of ER, therefore monitoring the focal Ca^2+^ concentration rather than the average concentration in the whole cell. In order to test the ability of ER-targeting N_3_-fura-2 to monitor focal Ca^2+^ concentration changes, cells were subjected to the stimulation of histamine, which can increase cytosolic Ca^2+^ level by mediating extracellular Ca^2+^ influx and ER Ca^2+^ release.[18] A Ca^2+^ level increase can be concluded from an increase in the ratio of F340 to F387, and vice versa.[19] Upon histamine stimulation, a sudden increase in F340/F387 ratio was observed (Figure 4(a)), indicating an increase in Ca^2+^ concentration in the region close to ER was monitored by ER-targeting N_3_-fura-2. To show Ca^2+^ response with the fine structures of ER, we focused on the fluorescence intensity of F387 which was greater than F340 in resting cells when the same imaging parameters were used in our setup. F387 exhibited a sudden decrease in intensity followed by a gradual recovery in response to histamine stimulation (Figure 4(b)). F387 intensity has a negative correlation with Ca^2+^ concentration.[20] Therefore, the fluorescence intensity changes of F387 corresponded to a sudden increase in Ca^2+^ level followed by a gradual drop, which was consistent with the result indicated by the ratio data (Figure 4(a)).

The response of the Ca^2+^ sensors on ER membranes would provide more precise information in judging the involvement of Ca^2+^ channels on ER membranes in a drug-elicited cytosolic Ca^2+^ increase when compared with the response of cytoplasm-localized Ca^2+^ sensors that detect the average cytosolic Ca^2+^ concentration. Furthermore, the Ca^2+^ sensors on ER membranes could be used for direct visualization of the heterogeneous distribution of these Ca^2+^ channels.[21,22] ER subdomains enriched with Ca^2+^ channels may release more Ca^2+^ and create a higher focal Ca^2+^ concentration than the subdomains with fewer Ca^2+^ channels in response to stimulus, resulting in heterogeneous responses of Ca^2+^ sensors at different subdomains. It is noteworthy that Ca^2+^ diffuse rapidly in cells and the gradients around channels disappear within a few hundred milliseconds.[20] Therefore, a high temporal resolution is required during image acquisition. And the trade-off between temporal resolution and fluorescence intensity should also be considered when setting up the acquisition parameters to ensure a sufficient signal to noise ratio is achieved.

## 4. Conclusions

In summary, we reported a method for specific and stable targeting of fluorescent organic dyes N_3_-fura-2 in living cells by scAVD–biotin interaction and copper-free click reaction. Using this method, we realized a prolonged retention period of N_3_-fura-2 in the cytosol of cells that is consistent with the previous report.[14] In addition, we successfully targeted N_3_-fura-2 to the cytosolic side of ER membranes. Thus, our study demonstrated the ability of specific targeting of traditional chemical Ca^2+^ indicators and generating Ca^2+^ sensors at specific subcellular organelles. In response to histamine stimulation, which increases cytosolic Ca^2+^ levels, ER-targeting N_3_-fura-2 demonstrated fluorescence intensity changes. These data showed the proof of concept of sensing focal Ca^2+^ concentration changes with specifically targeted Ca^2+^ sensors. Using azide-functionalized dyes with appropriate Ca^2+^ affinities, our method could generate specifically targeted Ca^2+^ sensors at other subcellular compartments, for investigating the Ca^2+^ signaling in different regions of cells.[23–26] Moreover, specifically targeted functional sensors for detecting other chemical and physiological parameters, such as pH and temperature,[12] could also be generated with respective azide-functionalized dyes for studying the establishment and regulation of those parameters in different organelles.[27–30] Our method is therefore a potential tool to develop a wide range of specifically targeted functional sensors for investigation of various localized cellular activities.

## Disclosure statement

No potential conflict of interest was reported by the authors.

## Funding

This work was funded by the Japan Society for the Promotion of Science (JSPS) KAKENHI [grant no. 26107717], the Waseda University Grant for Special Research Project [grant no. 2014B-314]
